# Increased Distress in Neurooncological Patients, a Monocentric Longitudinal Study: When to Screen Which Patient?

**DOI:** 10.3390/diseases12090217

**Published:** 2024-09-16

**Authors:** Franziska Staub-Bartelt, Julia Steinmann, Maren Wienand, Michael Sabel, Marion Rapp

**Affiliations:** 1 Department of Neurosurgery, University Hospital Düsseldorf, 40670 Düsseldorf, Germany; 2 Department of Anesthesiology, University Hospital Düsseldorf, 40670 Düsseldorf, Germany

**Keywords:** longtime data, psycho-oncological distress, brain tumour, malignant glioma

## Abstract

Objective: Neurooncological patients are well-known to experience an increased psycho-oncological burden with a negative impact on distress, therapy adherence, quality of life, and finally survival. But still, psycho-oncological screening and support is rare, with ongoing discussion about specific screening time points and impact factors. Therefore, we analysed the psycho-oncologic treatment demand at specific disease-related time points throughout therapy. Methods: In this longitudinal, prospective, single-centre study, patients with malignant brain tumours were screened for increased distress (using the Distress Thermometer), anxiety, depression (Hospital Anxiety and Depression Scale questionnaire), and health-related quality of life interference (EORTC QLQ C30-BN20 questionnaire) at specific longitudinal time points during therapy. The results were correlated with sociodemographic and clinical data. Results: From 2013 to 2017, 2500 prospective screening data points from 512 malignant brain tumour patients were analysed. DT was identified as a significant predictor for psycho-oncological treatment demand (*p* < 0.001). Particularly significant time points concerning psycho-oncological burden were primary diagnosis and tumour recurrence. Next to these known factors, here, patients < 65 years old and female patients (*p* = 0.018 and *p* = 0.017) reflected increased screening results, whereas partnership and professional activity (*p* = 0.043; *p* = 0.017) were identified as contributing factors to a significantly decreased treatment demand. Conclusions: The increased need for psycho-oncological support for neurooncological patients is underlined. Psycho-oncological support should particularly be offered at the time points of primary diagnosis and tumour recurrence. To support the positive effect of caregivers, they should be involved at an early stage.

## 1. Background

Malignant brain tumour patients are confronted with the burden of an incurable disease, which will inevitably lead to neurological and neuropsychological deficits and, finally, death. Therefore, during disease, patients may be withdrawn from their social life. As a consequence, the diagnosis of a brain tumour can cause fear and anger. Distress, anxiety, and depression are well-known comorbidities. Overall, in 35% of cancer patients, distress is reported regardless of the specific diagnosis [1]. Neurooncological patients are even more often affected by increased distress, with the literature reporting a prevalence of distress in up to 52% of patients [2,3] and depressive symptoms in about 21% of patients [4,5].

Increased distress and depression finally result in decreased patient compliance, leading to significantly worse general outcomes, especially in patients with high-grade gliomas. The overall survival (OS) of cancer patients, including HGG patients, is negatively affected by a significant correlation between a reduced health-related quality of life (HRQoL) and high levels of distress, anxiety, and depression [6,7,8,9,10].

During therapy, neurooncological patients are faced with different crucial events. Along with the time point of first diagnosis, patients may have to undergo surgical resection, radiotherapy, and chemotherapy, fearing tumour recurrence. Thus, specific therapy- and illness-related events during the course of disease are crucial and have to be identified. Recent studies have shown that heightened distress is often more likely to be observed at specific time points, such as during the perioperative phase [11]. However, still, to date, there is a lack of knowledge on how the psycho-oncological burden in brain tumour patients evolves. The recognition of these specific time points is crucial for healthcare providers to implement targeted interventions, such as preoperative counselling, enhanced communication, effective pain management strategies, and postoperative psychological support, in order to mitigate distress and improve patient outcomes during the perioperative phase.

Therefore, repeated psycho-oncological screening is highly relevant, as the implementation of professional support systems for patients in need is mandatory and their individual requirements might change over time. However, due to logistic reasons like changing medical departments during illness, fewer personnel in the treating hospital, and missing standardised neuropsychological screening tools, only a minority of the patients in need are diagnosed and referred to special support programs.

At our neurooncological department, standardised psycho-oncological screening has been established over recent years. In this study, we aimed to identify the contributing factors that might increase psycho-oncological burden and, therefore, increase the psycho-oncological treatment demand defined by specific cut-offs of screening instruments for malignant brain tumour patients at different time points. Furthermore, the impacts of sociodemographic and clinical data on psycho-oncological distress and treatment demand were assessed, as well as different aspects of QoL.

## 2. Patients and Methods

This prospective, single-centre, longitudinal analysis included patients that underwent psycho-oncological screening assessments between 2013 and 2017. The local ethical committee (study number 4087) approved the study. All patients enclosed in this study gave written informed consent.

Systematic data screening was performed during hospitalisation or at the outpatient department. Sociodemographic and clinical data were obtained from the local patient data management system (Medico, CompuGroupMedical, CGM Clinical Europe GmbH)

The inclusion criteria for this study were:

(1) Longitudinal treatment at our neurooncological-neurosurgical department;
(2) Age > 18 years at diagnosis;
(3) Neuropathological confirmed diagnosis of a malignant intracranial lesion;
(4) Ability to give written informed consent.

### 2.1. Timeline for Patient Survey

Patients were routinely followed up in three-month intervals at our outpatient clinic. The following specific illness-related time points were defined:

T0: perioperative after first diagnosis, T1: 3–6 months after diagnosis, T2: 12–24 months after diagnosis, T3: 24–36 months after diagnosis, and T4: >36 months after diagnosis to obtain long-term data during illness. As specific illness-related time points, the diagnosis of the first tumour recurrence (T0.1) and further tumour recurrences (T0.2) were emphasised.

At each time point, patients were informed about the suspected or final neuropathological diagnosis and further treatment strategies (following radio and/or chemotherapy).

### 2.2. Psycho-Oncological Screening Assessments

Distress, depression, and anxiety screening was performed based on self-assessment questionnaires using the Hospital Anxiety and Depression Scale (HADS) and the National Comprehensive Care Cancer Network, NCCN distress thermometer (DT). Additionally, the European Organization for Research and Treatment of Cancer (EORTC) QLQ-C30-BN20 was used to assess HRQoL issues [12,13].

#### 2.2.1. DT

According to the NCCN guidelines, the cut-off for a diagnosis of distress is a score of five or above, and psycho-oncological support is recommended. Besides the thermometer scale, the questionnaire also contains a list of 40 symptoms representing practical, family, emotional, spiritual–religious, and physical concerns.

#### 2.2.2. HADS

The *HADS* has been established as an effective screening tool for the assessment of anxiety and depression. It is a 14-item self-report questionnaire consisting of 7 items used to identify anxiety (HADS-A) and 7 items for depression (HADS-D), with each item using a four-point (0–3) Likert-type scale. For the subscales, a cut-off score of >eight is assumed to be optimal for patients [14,15,16]. The described cut-off values were also used in the present analysis.

In addition to the assessment of psycho-oncological distress, the results of the DT and HADS were also used to define treatment demands. Linear regression was performed to identify the impacts of single items on psycho-oncological treatment demand. Psycho-oncological treatment demand (DT and HADS) was used as the dependent variable and DT, fear, and depression (HADS), quality of life, global health status, and future uncertainty were used as explanatory variables. Furthermore, to evaluate different aspects of distress and their impacts on a potentially greater psycho-oncological treatment demand, the single items of the DT problem list were examined (physical, practical, family, emotional, and spiritual problems) in a multiple regression analysis.

The *EORTC QLQ-C30-BN20* was developed by the European Organization for Research and Treatment of Cancer (EORTC). The QLQ-BN20 is an additional module for brain tumour patients. The following sub-assessments were performed: global health, overall QoL, health-related quality of life (QoL2), and future uncertainty. The threshold for the global health and quality of life score was set to ≤4. For emotional and cognitive function, as well as future uncertainty, a cut-off ≥ 2.75 was applied according to the recommended scoring manual of the EORTC.

Additionally, the Karnofsky Performance Status (KPS) was assessed to quantify overall well-being and limitations in activities of daily living.

### 2.3. Statistical Analyses

Statistical analyses were conducted using IBM SPSS Statistics Version 24 (IBM Corporation, Chicago, IL, USA). Visualisation was performed using Microsoft Office Excel Version 2021 and Word for Mac Version 2021.

In the statistical analysis based on descriptive statistics, the influences of respective sociodemographic and clinical factors on the screening results were calculated using the Chi-square contingency analysis. The contingency coefficient was interpreted as being dependent when X2 = 0 and as independent when X2 > 0. The nature of the nominal-scaled relationship was interpreted using the Phi (Φ) coefficient according to Cohen as follows: small effect Φ = 0.1, medium effect Φ = 0.3, and large effect Φ = 0.5. Mean differences between the two groups were compared using the Welch test as an independent t-test for samples with heterogeneous variance. This approach allowed for the inclusion of a larger number of patients. For comparing individual groups, linear relationships were assessed using non-parametric Spearman rank correlations. The extent of the linear relationship was quantified with the correlation coefficient ρ = r (with a values ranging from −1 to +1) and interpreted according to Cohen. To identify the predictors and protective factors associated with psycho-oncological distress and to assess their influence and predictability, multiple regressions were conducted at all defined measurement points. The overall model and each regression coefficient were examined for statistical significance. Statistical significance was indicated by the *p*-value, with a significance level of α = 0.05 set for all tests.

## 3. Results

### 3.1. Patient Characterisation—Sociodemographic and Clinical Data Cohort Summary

Between 2013 and 2017, 2500 screening data points from 512 malignant brain tumour patients were identified (221 female (43%) and 291 (57%) male; mean age 55.9 years, range 19–86 years).

In 161 patients (31%), data only at a single screening time point could be obtained, with 99 patients (19%) participating at the time of initial diagnosis, 30 patients (6%) at the time of recurrence diagnosis, and 32 patients (6%) during the long term. A total of 351 patients (69%) participated in at least one follow-up screening, with a mean observation period of 20 months (range 1–59 months).

At T0, data were available from 276 patients (54%) and from 205 patients (40%) at T1, respectively. At T2, we could analyse data from 102 patients (20%), and data from 55 (11%) at T3 and 75 (14%) at T4. Overall, 203 patients (40%) suffered from recurrent tumour disease, 127 (63%) showed a first recurrence (T0.1), and 76 (37%) multiple recurrences (T0.2).

Neuropathological diagnosis was, due to the assessment time points, referred to the histopathological classification from 2016. In all patients, a malignant diagnosis was present. Therefore, all patients received further adjuvant treatment (radiotherapy, chemotherapy, or systemic therapy) according to the actual guidelines, depending on their diagnosis. In this cohort, 219 patients (43%) suffered from glioblastoma, 157 (31%) from anaplastic astrocytoma, and 136 (27%) from brain metastasis.

Active employment was reported for 16% of the patients at the time point of the survey, and more than one-third of the patients were pension recipients (35%). The majority of patients (309, 60%) had children and 324 (63%) were living in a marriage/life partnership.

In 119 patients (23%), a pre-existing psychiatric disorder was known. A positive psychiatric medical history was referred to in the case of the presence of any diagnosed mental health disorder, as classified by the International Classification of Diseases (ICD) system. This included any condition listed under the ICD codes within the range from F00 to F99, which covers mental, behavioural, and neurodevelopmental disorders. A positive history was identified when a patient had been previously diagnosed with one or more of these conditions by a healthcare professional, based on the criteria outlined in the relevant ICD code. Furthermore, 121 patients (24%) reported a positive medical history for the present or past consumption of sedatives. Sociodemographic cohort data and data on clinical findings are summarised in Table 1.

### 3.2. Treatment Demand Defined by DT and HADS—Screening Results from All Time Points

The DT revealed a high percentage of patients with noticeable distress (cut-off > 5) throughout the entire observation period (median 5.05). The highest values were generally observed at T0, T0.1, and T0.2. However, the obtained results showed no significant differences between DT at T0 and the course of disease. Concerning the evaluation of HADS, a low percentage of patients exhibited noticeable distress throughout the entire duration of the observation period (HADS-D median 6.69; HADS-A median 5.74). Descriptive analysis showed a significant increase in generalised anxiety and/or depression during therapy (T1, *p* < 0.018), as well as at diagnosis of tumour recurrence (T0.1, *p* < 0.007; T0.2, *p* < 0.005), and sub scores of HADS (HADS-A and HADS-D) showed no significant differences over the entire observation period. Table 2 summarises the results of the DT and HADS over the screening period.

In the linear regression model for the prediction of treatment demand, DT was found to be a significant predictor for psycho-oncological treatment demand during the entire observation period (T0: b = 0.72; t (196) = 13.89, *p* < 0.001; T1: b = 0.72; t (107) = 10.39, *p* < 0.001; T2: b = 0.64; t (57) = 7.65, *p* < 0.001; T3: b = 0.85; t (26) = 7.75, *p* < 0.001; T4: b = 0.82; t (19) = 5.91, *p* < 0.001; T0.1: b = 0.68; t (78) = 7.73, *p* < 0.001). Only at T0.2. was fear seen as an additional significant predictor (b = 0.28; t (47) = 2.19, *p* < 0.033).

Multiple linear regression with a DT cut-off score of ≥5 as a dependent variable and single items of the DT problem list as explanatory variables showed significant results at T0 (F(5, 192) = 4.84, *p* < 0.00), T1 (F(5, 109) = 4.29, *p* < 0.001), T2 (F(5, 60) = 6.47, *p* < 0.001), T3 (F(4, 27) = 6.10, *p* < 0.001), and T0.1 (F(5, 82) = 3,71, *p* < 0.004). Emotional problems were a significant predictor at all time points (T0: b = 0.07; t (47) = 2.97, *p* < 0.003; T1: b = 0.09; t (47) = 3.28, *p* < 0.001; T2: b = 0.12; t (47) = 3.86, *p* < 0.000; T3: b = 0.10; t (47) = 2.29, *p* < 0.030; T0.1: b = 0.10; t (47) = 3.55, *p* < 0.001). Spiritual problems were a significant predictor at T2 (b = 0.41; t (47) = 2.02, *p* < 0.0048), as were practical problems at T3 (b = 0.38; t (47) = 3.37, *p* < 0.002).

### 3.3. Impact Factors Regarding Treatment Demand

The Chi-square test revealed a significant relation between female gender and psycho-oncological treatment demand at T0 (X2(1) = 5.72, *p* < 0.017, j = −0.16) and T0.2 (X2(1) = 4.87, *p* < 0.027, j = −0.28). Between age (dichotomised < 65 years and ≥ 65 years) and psycho-oncological treatment demand, a significant relation for patients < 65 years was found at T0 only (X2(1) = 5.57, *p* < 0.018, j = −0.16). A positive relationship status correlated with a lower psycho-oncological treatment demand, with significant results being found at the time point of recurrence (T0.1; X2(1) = 4.09, *p* < 0.043, j = −0.21). Patients who were still working were significantly less burdened at T2 (X2(1) = 5.71, *p* < 0.017, j = −0.29) and tendentially also less burdened at T4, T0.1, and T0.2. A history of psychiatric treatment appeared to be a risk factor for psycho-oncological treatment demand at T0, T3, and T0.2. A significant correlation was only found at T1 (X2(1) = 5.04, *p* < 0.025, j = 0.21). A history of medical treatment with psychotropic drugs was a significant risk factor at T0 (X2(1) = 3.98, *p* < 0.046, j = 0.1) and T1 (X2(1) = 6.35, *p* < 0.012, j = 0.23), and was associated with a greater psycho-oncological treatment demand at every time-point. A lower postoperative KPS was significantly correlated with a greater psycho-oncological treatment demand at T1 (X2(1) = 3.88, *p* < 0.049, j = 0.17) and T2 (X2(1) = 4.12, *p* < 0.042, j = 0.24. In the recurrent setting, there was a trend in the same direction. Table 3 illustrates summarised results of the correlation between treatment demand (measured by DT and HADS) and sociodemographic and clinical data.

### 3.4. Psycho-Oncological Treatment Demand Correlating with HRQoL

The mean QoL score and psycho-oncological treatment demand (DT and HADS) only correlated significantly at T0.2 (r = 0.340, *p* < 0.010). A lower mean global health score was associated with a greater psycho-oncological treatment demand at every time point. Significant results were found at T0 (r = −0.30, *p* < 0.001), T1 (r = −0.40, *p* < 0.001), T2 (r = −0.46, *p* < 0.001), T0.1 (r = −0.33, *p* < 0.002), and T.0.2 (r = −0.30, *p* < 0.026). A higher mean score for future uncertainty was associated with a greater psycho-oncological treatment demand for the whole observation period. Significant correlations were found at T0 (r = 0.35, *p* < 0.001), T1(r = 0.29, *p* < 0.001), T2 (r = 0.57, *p* < 0.001), T0.1 (r = 0.38, *p* < 0.001), T3 (r = 0.39, *p* < 0.016), and T4 (r = 0.38, *p* < 0.031). Furthermore, the Chi-square test revealed a significant relation between a restricted HRQoL and psycho-oncological treatment demand at T0 (X2(1) = 7.06, *p* < 0.008) and T1 (X2(1) = 19.13, *p* < 0.001, j = 0.39). Future uncertainty was significantly influenced by a histopathological diagnosis of glioblastoma at T.0.1. (t (113, 27) = −2.57; *p* < 0.011).

### 3.5. Impact of Neurological Decline on Psycho-Oncological Treatment Demand and QoL

Regarding a reduced KPS, a trend of association with noticeable psycho-oncological distress was observed almost consistently over the entire observation period. The correlation was statistically significant during therapy after 3 to 6 months (T1, 0.003, X2(1) = 8.81, *p* < 0.003, φ = 0.25). Patients with reduced KPS showed a significant association with a reduced HRQoL and a high future uncertainty during therapy (T1: HRQoL: X2(1) = 4.10, *p* < 0.043, φ = 0.15; FU: X2(1) = 5.59, *p* < 0.018, φ = 0.17).

## 4. Discussion

According to the literature, 73% of neurooncologic patients present with increased distress, 15–38% with depressive disorders, and 30% with a prevalence of anxiety symptoms. Psycho-oncological symptoms are associated with reduced therapy compliance, shorter survival times, and higher suicide rates. Therefore, early diagnosis and intervention are essential, and knowledge on the risk and predictive factors for psycho-oncological stress is of crucial relevance for holistic patient care. However, despite this being known, the implementation of patient-reported outcome measurements (PROMs) in clinical daily routine is still missing—mainly due to time and personal restrictions [17]. To enable optimised assessments, which are time- and personnel-saving on the one hand, but still sensitive enough to identify patients at risk on the other hand, the identification of (a) sensitive time points in the course of therapy and (b) time-independent risk factors that negatively influence psycho-oncologic distress are of the utmost importance.

Here, we present—to the best of our knowledge—the first prospective longitudinal data analysis of >500 malignant brain tumour patients during therapy, including long-term data on >36 months of observation time.

### 4.1. Psycho-Oncological Distress during Therapy

Independent from the different time points, our data underline the increased psycho-oncological distress in malignant brain tumour patients [18,19,20,21,22,23,24,25].

In our study, the DT revealed increased levels of psycho-oncological distress in 63.4% (range: 54–76%) and HADS in 21.7% (range: 10–34%) of all patients.

PROMs were assessed at different longitudinal time points (after 6 (T1), 12 (T2), 24 (T3), and >34 (T4)) after first tumour diagnosis, reflecting a stable increased distress level during therapy, as well as anxiety and depression levels, with the lowest level occurring between 12 and 24 months after primary diagnosis (T2 and T3). Rooney et al. revealed that psycho-oncological burden even decreases over time in a longitudinal study over 6 months with 154 patients. As a potential explanation, a shift in terms of reappraisal and adjustment over time is discussed [8]. Mainio et al. and Pringle et al. showed a significant decrease in fear and depression during therapy [12,18], which might be explained by disease processing and acceptance.

Our hypothesis that disease-specific time points are crucial and sensitive in neurooncological patients can be confirmed by the highest observed prevalence rates for psycho-oncological distress at these time points, independent of the assessment tool. These results are of the utmost importance, underlining the impacts of disease-specific time points. In reverse, these data facilitate the time- and personnel-saving support offered to our patients, as these data raise awareness among medical staff about these sensitive time points.

Present studies assume a greater burden at recurrence (29). Trad et al. observed a prevalence of 37% for psycho-oncological distress at primary diagnosis and 75% at recurrence, with a significant increase in depression at the time point of tumour recurrence [13]. The treatment for malignant brain tumours is still palliative and patients live with the knowledge of inevitable tumour progression. The objective confirmation of this event, for example, by the diagnosis of tumour recurrence in the MRI follow-up scan turns this theoretical knowledge into a realistic threat.

The level of unspecific psycho-oncological distress measured by the DT was higher throughout the whole observation period than the level of specific stress caused by fear and depression (HADS), which is described in the current literature [12,14,15,16]. In our study, distress (DT) was found to be a significant predictor for psycho-oncological treatment demand throughout the entire observation period. For further specification, it is possible to add the results of the DT problem list to increase the specificity and sensitivity of DT assessments [19]. On the one hand, this combination facilitates assignment to subspecialties, e.g., oncology, psychology, and social work [20]. On the other hand, it makes screening more time-consuming and more difficult to evaluate. Here, further specification with the DT problem list revealed emotional problems as a significant predictor. Therefore, we recommend—if possible—to focus on this item in routine psycho-oncological screening.

### 4.2. Sociodemographic and Clinical Impact Factors for Increased Psycho-Oncological Distress

Psychiatric disorders and increased neurological symptoms were found to be the most important independent risk factors [19,21] in most studies. Furthermore, comorbidities, female gender, a young age, histopathological diagnosis, and familiar and emotional problems have been discussed as risk factors [14,19,22,26].

In our study, next to the first known impact factors (psychiatric disorders and neurological disorientation), significant risk factors for psycho-oncological treatment demand were female gender and a younger age (<65 y). Women are known to be a vulnerable group in cancer care [19,22,23]. Potential reasons for this are gender roles and social norms. Still, in our society, women are mainly caregivers, keeping the familial structure together. Finding themselves in the position of a person who needs help can, therefore, cause additional stress by changing the family role distribution. Younger patients often have fewer comorbidities, but the diagnosis of a brain tumour changes their whole life considering their social roles. Additionally, younger patients may have increased financial burdens [8,18,19] caused by the role of being the breadwinner of the family.

Which factors may reduce distress and, therefore, need to be supported by medical staff? Here, during therapy, two factors mainly reflected a positive impact, relationship and professional activity.

For most people, a relationship means support and resilience. Couples possibly develop strategies over time to adapt to their situation and overcome initial problems. These results concur with Arnold et al., who observed that married patients are less burdened. On the other hand, this means significantly increased distress for the caregivers themselves. Multiple studies have underlined that the caregivers of cancer patients see a significant increase in the prevalence of psychosocial burden. There are emotional but also practical reasons for this, e.g., taking care of everyday challenges. Worries about the future and sorrow of their caregivers are a relevant burden for cancer patients [24]. Recent studies confirmed a mutual influence for caregivers and patients. Therefore, it is important to include caregivers in psycho-oncological support.

Professional activity is a protective factor during disease [16,21,23]. Psycho-oncological treatment should, therefore, also consider the preservation of or reintegration into professional activity.

### 4.3. Psycho-Oncological Treatment Demand, Quality of Life, Future Uncertainty, and Global Health Status

Quality of life and future uncertainty are important parameters to further differentiate psycho-oncological distress [15,25,27,28]. Hoffmann et al. showed correlations between perioperative psycho-oncological distress and a decreased quality of life, global health status, and greater amount of future uncertainty [25]. QoL was found to be an independent predictor for therapy adherence, survival, and suicidality in patients with brain tumours [29,30]. In our study, psycho-oncological distress correlated significantly with HRQoL, future uncertainty, and global health status in the first year of disease. Furthermore, a significant correlation was found between future uncertainty and global health status at recurrence. Our results are consistent with the literature [14,23,31,32,33]. The screening results on HRQoL, future uncertainty, and global health status reflect a greater burden in patients with malignant brain tumours, which is accompanied by a greater psycho-oncological treatment demand [31,34].

### 4.4. Study Limitations

Data were collected in clinical daily routine. In total, 31% of all patients were only screened at one time point. Consequently, no individual longitudinal statistical analysis was possible. We chose important time points during the disease course. To obtain a relevant number of patients for each point, we had to combine follow-ups. Hereby, we possibly missed time points with changes in psycho-oncological distress. Due to the poor prognoses of patients with glioblastomas, the sample sizes became significantly smaller from T3 to T0.2. This made comparability between time points more difficult. The DT was completed more frequently than the HADS for all time points. This limits the reliability of our results. The guidelines for psycho-oncological screening recommend adding the individual question for treatment demand to screening tools. In our study, psycho-oncological treatment demand was only determined by DT and HADS. Furthermore, we included all malignant intracranial lesions, recognising the wide variability among malignant tumours in terms of their histological types, growth patterns, and clinical outcomes. This heterogeneity could influence our findings, as different tumour types may respond differently to treatments and exhibit distinct patterns of progression. Consequently, our broad inclusion criteria may have introduced variability into the data, potentially obscuring specific effects or trends related to particular tumour subtypes. Future studies with more homogeneous samples or stratified analyses by tumour type may be necessary to clarify these effects and provide more targeted insights.

### 4.5. Clinical Implication

Our data report the results of psycho-oncological distress in patients with malignant gliomas, (1) presenting the DT as a time- and personnel-sparing assessment tool to identify patients at risk and (2) emphasising sensitive time points in the course of therapy when patients are more affected by distress (primary diagnosis and tumour recurrence), on the one hand identifying patient groups at risk and, on the other hand, resources that may decrease psycho-oncological distress.

## 5. Conclusions

Malignant glioma patients should be screened for increased psycho-oncological distress to enable timely support. Medical staff should be sensitised at the time points of first diagnosis and tumour recurrence. Our data suggest that patients who require initial support will need it more over time, while other patients who require less support will not necessarily need it throughout therapy.

## Figures and Tables

**Table 1:** Patient Characteristics—sociodemographic and clinical data of included patients at all different time points.

	All Patients	Primary Diagnosis	First Recurrence	Multiple Recurrence
**n**	512	417	164	74
**gender**				
female	221 (43%)	237 (57%)	99 (60%)	40 (54%)
male	291 (57 %)	180 (43%)	65 (40%)	34 (46%)
**age**				
median	56	58	54	49
range	19–86	19–86	23–81	28–76
<65 years	365 (70%)	277 (66%)	130 (79%)	65 (88%)
>65 years	156 (30%)	140 (33%)	34 (21%)	9 (12%)
**relationship status**				
partnership	324 (63%)	257 (62%)	117 (71%)	57 (77%)
single	97 (19%)	84 (20%)	32 (20%)	12 (16%)
not specified	91 (18%)	74 (18%)	15 (9%)	5 (7%)
**children**				
yes	309 (60%)	267 (64%)	100 (61%)	43 (58%)
no	110 (21%)	84 (20%)	48 (29%)	26 (35%)
not specified	93 (19%)	76 (16%)	16 (10%)	5 (7%)
**professional status**				
employed	80 (16%)	70 (17%)	30 (18%)	11 (15%)
on sick leave	119 (23%)	101 (24%)	48 (29%)	17 (24%)
pension recipient	179 (35%)	139 (33%)	54 (33%)	34 (45%)
not specified	93 (26%)	107 (26%)	32 (20%)	12 (16%)
**Diagnosis (WHO 5)**				
anaplastic astrocytoma	157 (31%)	105 (25%)	57 (35%)	33 (45%)
glioblastoma	219 (43%)	192 (46%)	76 (46%)	25 (34%)
metastasis	136 (26%)	120 (29%)	31 (19%)	16 (21%)
**comorbidities**				
yes	220 (43%)	190 (46%)	68 (41%)	30 (41%)
no	317 (38%)	149 (36%)	79 (49%)	38 (51%)
not specified	95 (19%)	78 (18%)	17 (10%)	6 (8%)
**positive psychiatric medical history**				
yes	119 (23%)	86 (21%)	48 (29%)	28 (38%)
no	317 (62%)	270 (65%)	101 (62%)	42 (57 %)
not specified	76 (15%)	61(14%)	15 (9%)	4 (5%)
**positive psychotropic drug history**				
yes	121 (24%)	89 (21%)	42 (26%)	22 (30%)
no	308 (60%)	261 (63%)	105 (64%)	48 (65%)
not specified	83 (16%)	67 (16%)	17 (10%)	4 (5%)

**Table 2:** Differences in psycho-oncological distress measured by DT and HADS during disease. Mean values are stated. Bold numbers are significant, level of significance is indicated by asterisk operators.

Screening Parameter	T0	T1	t	T2	t	T3	t	T4	t	T0.1	t	T0.2	t
DT	5.35	4.88	1.66	4.81	1.52	4.74	1.32	4.91	0.88	5.44	0.29	5.97	1.66
HADS-T	9.65	10.82	1.47	10.5	0.79	8.38	1.03	11.15	1.02	11.33	1.79	14.97	4.2
HADS-A	4.53	5.65	2.39 *	5.36	1.46	3.74	1.18	5.27	0.97	5.97	2.74 **	7.83	4.75 ***
HADS-D	5.14	5.17	0.07	5.29	0.25	4.64	0.75	5.88	0.89	5.36	0.44	7.16	2.86 **

t-test significance * *p* < 0.05 ** *p* < 0.01 *** *p* < 0.001. T0: perioperative after first diagnosis T1: 3–6 months after diagnosis, T2: 12–24 months after diagnosis, T3: 24–36 months after diagnosis and T4: >36 months after diagnosis to obtain long-term data during illness. As specific illness-related time points, the diagnosis of the first tumour recurrence (T0.1) and further tumour recurrences (T0.2) were emphasized.

**Table 3:** Correlation between treatment demand (DT and HADS) and sociodemographic and clinical data throughout the disease course. Bold numbers are significant, level of significance is indicated by asterisk operators.

Screening Parameter	T0	T1	T2	T3	T4	T0.1	T0.2
**DT, HADS & Gender**							
Chi-Square	5.72 *	1.86	0.07	0.45	2.39	0.63	4.87 *
Phi	−0.16	−0.12	−0.03	0.11	−0.26	0.08	−0.28
**DT, HADS & Age**							
Chi-Square	5.57 *	0.08	0.10	0.85	0.17	0.25	0.00
Phi	−0.16	0.02	−0.04	−0.15	0.07	0.05	0.00
**DT, HADS & relationship**							
Chi-Square	2.78	2.36	0.02	1.02	0.78	4.09 *	3.18
Phi	−0.12	−0.15	−0.02	−0.17	−0.15	−0.21	0.00
**DT, HADS & professional status**							
Chi-Square	0.23	0.37	5.71 *	0.00	0.89	2.74	1.48
Phi	−0.03	−0.06	−0.29	0.01	−0.16	−0.17	−0.16
**DT, HADS & somatic concomitant disease**							
Chi-Square	0.50	3.88 *	0.47	0.76	0.01	0.01	0.05
Phi	−0.05	0.19	0.08	−0.14	−0.02	0.01	−0.03
**DT, HADS & psychiatric medical history**							
Chi-Square	3.67	5.04 *	0.00	0.96	0.00	0.01	1.31
Phi	0.13	0.21	0.01	0.16	0.01	−0.01	−0.15
**DT, HADS & psychotropic drug history**							
Chi-Square	3.98 *	6.35 *	1.02	1.54	3.28	0.93	0.04
Phi	0.14	0.23	0.12	0.20	0.31	0.10	−0.03
**DT, HADS & KPS**							
Chi-Square	0.46	8.81 **	0.95	1.05	2.36	2.06	0.07
Phi	0.05	0.25	0.11	0.16	0.26	0.15	0.03

significance * *p* < 0.05 ** *p* < 0.01.

## Data Availability

Original data are available on request.
